# Learning from intersectoral initiatives to respond to the needs of refugees, asylum seekers, and migrants without status in the context of COVID-19 in Quebec and Ontario: a qualitative multiple case study protocol

**DOI:** 10.1186/s12961-023-00991-x

**Published:** 2023-06-20

**Authors:** Lara Gautier, Erica Di Ruggiero, Carly Jackson, Naïma Bentayeb, Marie-Jeanne Blain, Fariha Chowdhury, Serigne Touba Mbacké Gueye, Muzhgan Haydary, Lara Maillet, Laila Mahmoudi, Shinjini Mondal, Armel Ouffouet Bessiranthy, Pierre Pluye, Saliha Ziam, Nassera Touati

**Affiliations:** 1 School of Public Health, University of Montreal, Suite 3076, 7101 Av du Parc, Montreal, QC H3N 1X9 Canada; 2 Centre de recherche en Santé Publique (CReSP), University of Montréal and CIUSSS du Centre-Sud-de-l’Île-de-Montréal, Montréal, Canada; 3 Dalla Lana School of Public Health, University of Toronto, Toronto, Canada; 4 SHERPA University Institute, CIUSSS West Central Montreal, Montreal, Canada; 5 Centre de recherche et de partage des savoirs InterActions, Université de Montréal, Montreal, Canada; 6 School of Rehabilitation Sciences, McMaster University, Hamilton, Canada; 7 Université du Québec en Abitibi-Témiscamingue (UQAT), Sherbrooke, Canada; 8 Université du Québec à Montréal (UQÀM), Montreal, Canada; 9 École Nationale d’Administration Publique, Montreal, Canada; 10 McGill University, Montreal, Canada; 11 École des Sciences de l’administration, Université TÉLUQ, Montreal, Canada

**Keywords:** Refugees, Asylum seekers, Migrants without status, Intersectoral collaboration, Community organizations, Public service providers, COVID-19

## Abstract

**Background:**

Refugees, asylum seekers, and migrants without status experience precarious living and working conditions that disproportionately expose them to coronavirus disease 2019 (COVID-19). In the two most populous Canadian provinces (Quebec and Ontario), to reduce the vulnerability factors experienced by the most marginalized migrants, the public and community sectors engage in joint coordination efforts called intersectoral collaboration. This collaboration ensures holistic care provisioning, inclusive of psychosocial support, assistance to address food security, and educational and employment assistance. This research project explores how community and public sectors collaborated on intersectoral initiatives during the COVID-19 pandemic to support refugees, asylum seekers, and migrants without status in the cities of Montreal, Sherbrooke, and Toronto, and generates lessons for a sustainable response to the heterogeneous needs of these migrants.

**Methods:**

This theory-informed participatory research is co-created with socioculturally diverse research partners (refugees, asylum seekers and migrants without status, employees of community organizations, and employees of public organizations). We will utilize Mirzoev and Kane’s framework on health systems’ responsiveness to guide the four phases of a qualitative multiple case study (a case being an intersectoral initiative). These phases will include (1) building an inventory of intersectoral initiatives developed during the pandemic, (2) organizing a deliberative workshop with representatives of the study population, community, and public sector respondents to select and validate the intersectoral initiatives, (3) interviews (*n* = 80) with community and public sector frontline workers and managers, municipal/regional/provincial policymakers, and employees of philanthropic foundations, and (4) focus groups (*n* = 80) with refugees, asylum seekers, and migrants without status. Qualitative data will be analyzed using thematic analysis. The findings will be used to develop discussion forums to spur cross-learning among service providers.

**Discussion:**

This research will highlight the experiences of community and public organizations in their ability to offer responsive services for refugees, asylum seekers, and migrants without status in the context of a pandemic. We will draw lessons learnt from the promising practices developed in the context of COVID-19, to improve services beyond times of crisis. Lastly, we will reflect upon our participatory approach—particularly in relation to the engagement of refugees and asylum seekers in the governance of our research.

## Background

Since 2020, severe acute respiratory syndrome coronavirus 2 (SARS-CoV-2)—which causes COVID-19—has caused a world-wide pandemic and put a spotlight on many of the pre-existing social and economic inequalities that are pervasive in society [1, 2]. Previous pandemics have taught us that infectious diseases have the strongest impact on marginalized groups due to the often higher exposure to vulnerability factors (e.g., substandard housing and working conditions) by these groups [3]. As a result of additional vulnerability factors (e.g., lack of permanent residence status) experienced by migrant populations, this population represents some of the most marginalized groups [4]. The literature suggests that the COVID-19 pandemic has disproportionately affected migrant populations across a range of socioeconomic and health outcomes, including a heightened risk of infection, worsened mental health, interrupted immigration processes, and increased challenges in access to health and social services and support resources [5–9]. Moreover, several categories of migrants, such as refugees, asylum seekers, and migrants without status, have been more affected by the pandemic than others [10, 11]. We suggest an intersectoral response is necessary to reduce the vulnerability factors experienced by the most marginalized migrants.

### The value of intersectoral collaboration

The concept of “intersectorality” suggests that improving population health requires the collaboration (i.e., coordinated efforts) of different sectors of society (e.g., communities, local governments, etc.) [12, 13]. Crucially, intersectorality requires a reframing of values away from health as a superseding goal toward promoting equity as an overarching goal. In doing so, the concept of intersectorality seeks to ensure equality between the diverse partners of such collaborations, through valuing and promoting the equal contribution of these different sectors of society [14].

There is evidence that collaborations between public health and healthcare organizations with community-based organizations (CBOs)—that go beyond the mere referral of patients to healthcare services—significantly enhance access to services for marginalized populations [15]. Intersectorality is also considered a “best practice” in response to pandemics because, when CBOs have direct communication links to public authorities, public health messages are swiftly conveyed as CBOs have closer access to underserved populations, thereby reducing the risk of infection in these population groups [16]. For example, in the USA, during the H1N1 outbreak in 2009, organizations serving Latino-American migrant and seasonal farm worker communities successfully communicated key information from the US Centers for Disease Control and Prevention. The proximity of CBOs to these communities was successfully leveraged through daily emails to key resource persons, such as migrant health center chief executive officers and migrant health clinicians, to ensure that the information quickly reached the intended populations. They also designed bilingual patient education tools that were transmitted to diverse migrant networks [16].

However, despite the above example, there is little research on the development of intersectorality during crises [17, 18]. The present research intends to fill this research gap. Indeed, across the globe, the start of the COVID-19 pandemic acted as a catalytic event, spurring the emergence of new intersectoral initiatives in response to increasingly diverse migrants’ needs [19].

### Migration categories and vulnerabilities

Through this research, we are particularly interested in migrants who have experienced cumulative vulnerabilities as they are the ones who express the highest need for health and social intervention [20–22]. Three migrant categories are considered most vulnerable (Table1): migrants without status, asylum seekers, and refugees. Migrants without status are mostly—in the Canadian context—persons who reside in the host country after their temporary permit has expired. Asylum seekers are persons who, upon entry or during a temporary stay, seek the protection of another government. Refugees and asylum seekers are persons forced to flee their home country in order to escape persecution, violence, or war.

**Table 1:** Three categories of vulnerable migrants in the Canadian context

Migrants without status	Persons who remain in the host country after their temporary permit has expired (e.g., student permit or temporary work permit)
Asylum seekers (also sometimes called “refugee claimants”)	Persons who, upon entry or during a temporary stay, seek the protection of another government. After a lengthy application process, they may or may not obtain refugee status
Refugees	Persons who were forced to flee their country in order to escape danger. In Canada, whether they are privately sponsored or government-assisted, refugees are admitted to the country with permanent resident status. Those who are recognized in Canada also eventually obtain permanent residence

The limited space for the present manuscript compels us to provide “simplified” definitions. We indeed acknowledge the complexity of these categories, as well as the diversity of people’s situations within each category—most particularly, the diverse situations of migrants without status

There are multiple data gaps on the health and wellbeing of these populations in Canada. The most vulnerable and underserved categories—migrants without status and asylum seekers—are the least documented in the literature. In Canada, government-assisted refugees are admitted with permanent resident status, granting them access to public services, community services, and financial aid. Privately sponsored refugees are also admitted as permanent residents, but their access to services and public financial aid may be limited [23]. Given the temporary or non-status nature that asylum seekers and migrants without status face, these individuals not only have reduced access to services, but they also face significant uncertainty, increasing their vulnerability (e.g., for asylum seekers, the uncertainty about the ability to obtain permanent residence, and for migrants without status, the uncertainty in seeking health and social services for fear of being turned away or being reported to authorities) [24, 25]. Our population of interest therefore includes a heterogeneous group of migrants who have very different circumstances. Given the limited data, our study will be a welcome addition to the literature as it will document differences of service experience across these three categories.

In Canada, public service providers aim to support all migrants benefiting from permanent residence; however, there are multiple barriers to service access (e.g., discrimination, delay of care due to a mandatory 3-month waiting period, complexity of the health system) [26]. CBOs that receive public funding from the municipal, provincial, and/or federal governments are officially mandated to deliver “certain” services (e.g., food security and employment assistance) to facilitate the integration of refugees (for Quebec), or “all” services necessary to facilitate their integration, ranging from orientation to housing and employment (e.g., in Ontario and most other Canadian provinces). These services are primarily accessible to refugees and, to a much lesser extent, to asylum seekers. For instance, in Quebec, certain CBOs were mandated to provide diverse services only to government-assisted refugees. Only recently were housing assistance services extended to asylum seekers in Quebec. The impetus falls on other (im)migrant-serving community-based and nongovernmental organizations to offer temporary housing, food, psychosocial support, healthcare, language classes, professional training, and legal and employment assistance as a complement to, and sometimes instead of, public organizations, especially for migrants lacking government support (e.g., migrants without status).

Recent studies have highlighted that, despite the availability of these diverse sources of support, migrants without status and some migrants with temporary status, experience poorer self-perceived health and more unmet health needs than Canadian citizens and economic immigrants [24, 27–29]. With many of the aforementioned services closing during the pandemic, these issues were further exacerbated [30, 31].

### Impacts of the pandemic on migrants’ physical and mental health

Health data on refugees, asylum seekers, and migrants without status are scarce in Canada [32]. The pandemic did not improve this situation due to the fact that data regarding migration status were not systematically collected in relation to COVID-19 infection, hospitalization, or mortality [30]. For this reason, we are extending our review of the consequences of COVID-19 beyond the aforementioned three population categories, to include other categories of migrants (e.g., seasonal migrants with a temporary status, and economic immigrants who have a permanent status), although we acknowledge the limitation associated with the differences in circumstances [33].

Ecological studies have shown that the interaction between race, socioeconomic status, and occupational status resulted in an increased risk of infection and excess mortality [34–37]. Migrants without status and other vulnerable categories of migrants may be particularly at risk for severe COVID-19 symptoms given the prevalence of (1) undiagnosed diseases and health conditions that remain untreated, and (2) comorbidities in this population (e.g., cardiovascular disease, hypertension, hepatitis B and C) [38]. In Canada, available studies suggest that refugees, asylum seekers, and migrants without status are at increased risk of contracting COVID-19 given their precarious living conditions (e.g., living in crowded housing with potentially dysfunctional ventilation systems, shared bathroom facilities, multigenerational housing, etc.) and working conditions (e.g., working as essential workers, for example as orderlies in long-term care facilities, who often have the status of asylum seekers) [39–41]. A growing body of evidence based on surveillance data indeed indicates that COVID-19 cases in large Canadian cities have been disproportionately concentrated in areas with a higher proportion of visible minorities, recent immigrants, high-density housing, and essential workers—all interrelated risk factors for refugees, asylum seekers, and migrants without status [42, 43]. In addition, the implementation of COVID-19 policies has exacerbated other vulnerability factors (i.e., employment loss, mistrust of institutions, barriers to healthcare access, etc.) further deteriorating their health [44] and leading to an even greater risk for infection. Statistics Canada data from late 2020 showed that recent immigrants (which includes refugees), and particularly those who have been in the country for less than 5 years, have poorer self-perceived mental health since the pandemic started: 28% of recent immigrants reported fair or poor self-rated mental health, compared with 20% of long-term immigrants and 24% of Canadian-born people [9]. Similarly, recent immigrants who were financially affected by the pandemic had higher levels of anxiety (21%) than the other two categories (11%) [9].

The present research focuses on Ontario and Quebec, which are Canada’s most populous provinces and are the two Canadian provinces that receive the larger share of refugees, asylum seekers, and migrants without status [45]. Studies from Quebec and Ontario demonstrate the disproportionate risk of infection for migrant populations compared with the general population [46–48]. More specifically, a 2020 Ontario report indicates that, while economic immigrants, refugees, and other migrants make up 25% of the province’s population, they accounted for 43.5% of the total number of COVID-19 cases during the first wave [46]. While rates of testing among this group were generally lower, among those tested, refugees tended to have the highest percent positivity rate (10.4% versus 7.6% in migrants with permanent status and 2.9% in Canadian-born and long-term residents) [46].

Research in metropolitan areas of both provinces have shed light on numerous difficulties experienced by migrant groups since the start of the pandemic. In Toronto, the rate of COVID-19 was 923 cases per 100,000 in immigrants, refugees, and other recent health insurance cards (OHIP) registrants, while for Canadian-born and long-term residents the rate was 565 COVID-19 cases per 100,000. The proportion of positive tests was higher in immigrants (6.1%), refugees (9.6%), and recent OHIP registrants (5.9%) in comparison with Canadian-born and long-term residents (2.8%) [46]. A Montreal study pointed out that (1) migrants with temporary status or without status had encountered many difficulties in accessing COVID-19 testing, and (2) several reports were made of working conditions that did not comply with health regulations in companies employing migrants without status [44]. Acknowledging these issues, in a key report on the future of public health post-COVID, Canada’s Chief Public Health Officer highlighted the importance of coordinated responses by working collaboratively with all levels of government and key stakeholders [49]. By analyzing the development and implementation of promising intersectoral initiatives to meet the diverse needs of refugees, asylum seekers, and migrants without status, the present project is directly in line with these public health recommendations.

### Impacts of the pandemic on intersectoral services for refugees, asylum seekers, and migrants without status

Enhancing intersectoral action is a major lever in the response to pandemics [16]. Community mobilization, as a pillar of intersectoral action, is an essential instrument for pandemic responsiveness [50–52]. As shown in a peer-reviewed rapid review by Loewenson and colleagues (2021), CBOs play a pivotal role in implementing solidarity- and equity-driven public health interventions that extend beyond the usual risk communication strategies (as described below) [53]. More evidence is needed to document how CBOs adapt and transform their actions in the context of health crises. Indeed, while public health restrictions to contain and slow the spread of COVID-19 changed the functioning of public service providers, these restrictions also significantly affected the actions of CBOs. A US report highlights the strong pressure (notably due to significant staffing shortages) exerted on CBOs assisting migrants during the pandemic, which reduced access to support services [54]. In Quebec and Ontario, CBOs reacted by adapting their practices, offering additional services, and switching to remote forms of support [17]. CBOs played a pivotal role in both infection prevention (i.e., by raising awareness and providing access to reliable, multilingual information on COVID-19) and social protection (i.e., by centralizing data on the availability of food aid, psychosocial support, screening, and access to healthcare related to COVID-19) [50, 55, 56]. It is essential to learn from both the community sector and the public sector who implemented promising initiatives during the pandemic with a specific focus on the needs of migrants to better prepare for future health crises [57], and to sustain the initiatives that may be considered promising from a health systems responsiveness perspective and by all research partners—from funders, policymakers, and service providers to migrant service users.

Using the Canadian case, and by applying a theory-informed and unique participatory approach through which migrants participate in the governance of our research project, we will highlight the lessons learnt from the implementation of promising initiatives that mobilized unprecedented forms of intersectoral collaboration between public and community actors.

## Methods/design

### Research aim

Our research objective is to analyze the implementation processes of intersectoral initiatives in the pandemic context, and to draw lessons for a sustainable response to the needs of refugees, asylum seekers, and migrants without status in two major Canadian sanctuary cities for migrants, i.e., Montreal and Toronto, and a smaller-size city with a history of welcoming refugees, i.e., Sherbrooke. We will be focusing on refugees, asylum seekers, and migrants without status who have arrived in Canada recently (i.e., between 2016 and 2021) to reflect upon the challenges of integration in the context of the COVID-19 pandemic.

To achieve this objective, we will engage in four activities. First, we will review intersectoral initiatives already documented in the literature and compile those in an inventory. Second, we will examine the implementation of the most promising intersectoral initiatives from the perspectives of community and public service providers, in the context of the pandemic (organizational and individual levels of analysis). Third, we will analyze the experiences of these services or lack thereof for refugees, asylum seekers, and migrants without status (individual level of analysis). Fourth, we will facilitate discussions regarding issues surrounding implementation, sustainability, and scaling-up of intersectoral initiatives (organizational and macropolitical levels of analysis).

### Research approach—participatory governance

This project builds on our research teams’ participatory experiences in engaging and recruiting knowledge users [58] (i.e., refugees, asylum seekers, CBOs, public organizations, policymaking bodies) as partners at all stages of the research and as co-creators of research products [59]. We will establish two advisory boards (one per province) whose membership will include refugees and asylum seekers (ten in total),^1^ public and community sector frontline workers and managers (ten in total) and city/provincial/federal policymakers (ten in total). The advisory boards will meet four times a year to contribute to all stages of the research. Refugees and asylum seekers will be recruited to sit on these boards through partnerships with CBOs at the beginning of the research. To enable these users to make the most of their role in the research process, our research team will provide orientation sessions based on existing guidelines for citizen engagement in research [60]. The advisory boards will ensure a two-way relationship and a common understanding between researchers and knowledge users, thereby accounting for knowledge users’ needs and expectations throughout the research process. This approach also allows for continuous quality assessment of processes, thus reinforcing criteria of methodological rigor, such as the reliability (internal validity) and transferability (external validity) of results [61]. While the theoretical model below will be used to frame the research and design data collection tools, methods and engagement processes will remain flexible.

### Theoretical model

This research is in line with the theory of complex adaptive systems (CAS), which has been used in crisis management research [62, 63]. CAS theory has notably been used to analyze health systems’ adaptations to migrant needs [64]. Furthermore, health systems are considered to be CAS [65]. In Canada, the term “health and social services system” is regularly used to recognize the intersectoral nature of the system. CAS are open to their environment, meaning the system responds to other organizations that are both within and external to the system. Each organization depends on and adapts to the environment (including external shocks such as pandemics), while at the same time demonstrating autonomy and learning capacities to act upon that environment [66, 67]. The “co-evolution” process refers to the interdependent relationship between an organization and its environment. According to Baum and Singh, co-evolution “assumes that changes may occur in all interacting populations or organizations, permitting change to be driven by both direct interactions and feedback from the rest of the system” [68]. In the context of intersectoral action, co-evolution can be defined as follows: “the different actors [of organizations and their environment], linked and acting in relation to each other, share a vision and the meaning of their actions is moving toward the same goal: adapting services to a sub-group with its own specificities” [69]. A shock, like the COVID-19 pandemic, reconfigures actors’ actions and relations; it may become even more difficult to “co-evolve” toward a shared objective, and the feedback system may malfunction due to predominant top-down decision-making. The voice of targeted service users—in this case, refugees, asylum seekers, and migrants without status—may no longer be heard. Inspired by CAS theory, the concept of “health systems responsiveness” starts with the idea that meeting people’s needs and expectations is an essential aspect of a system’s ability to withstand shocks and crises. Through this concept and its components (see below), we will examine the implementation of the selected intersectoral initiatives.

**Fig. 1:**
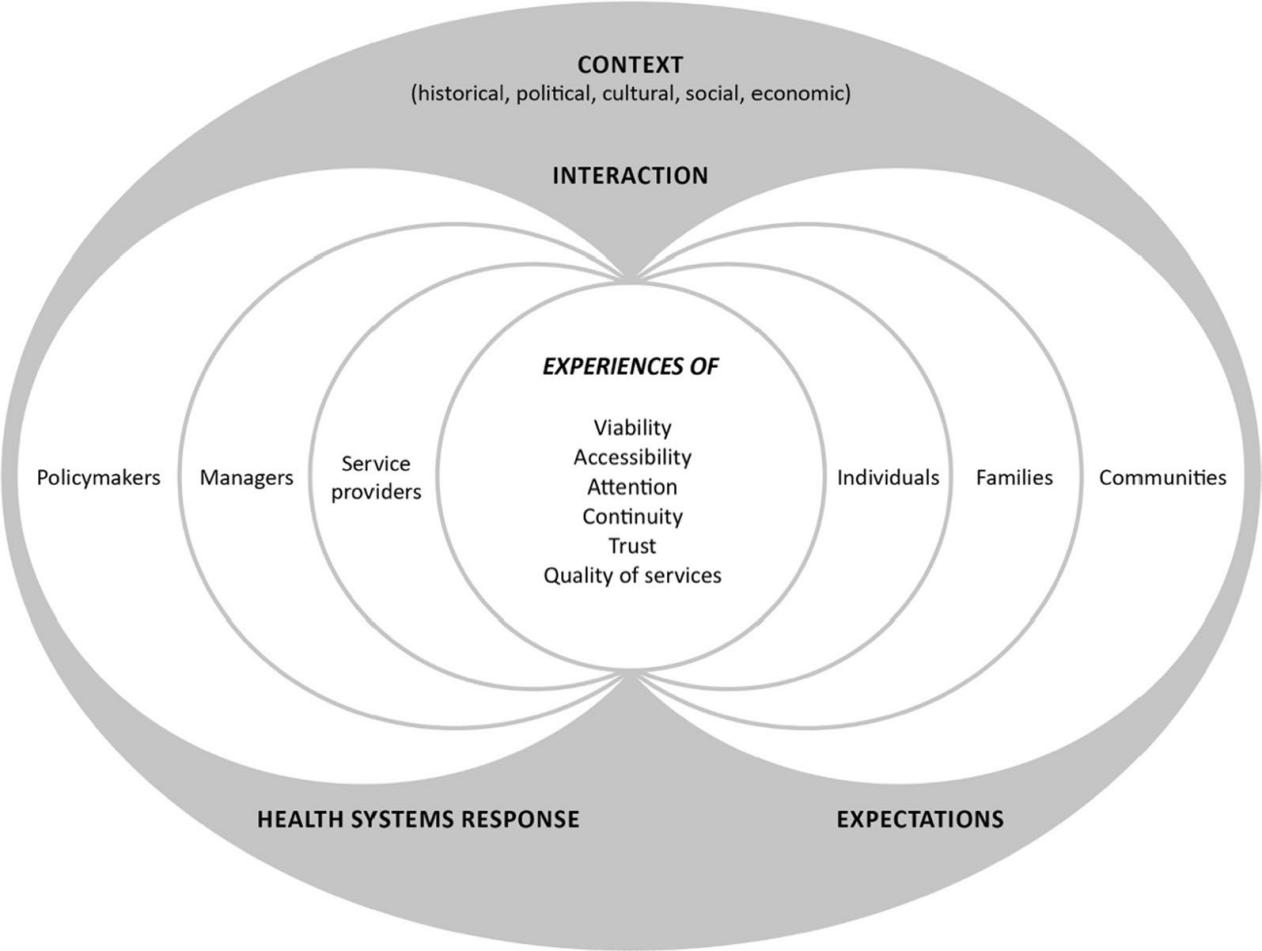
Framework for health systems responsiveness, adapted from Ref. [72, 73]

The concept of “health systems responsiveness” has also been applied outside the health sector [70] and is particularly relevant to the context of community and social care provision to refugees, asylum seekers, and migrants without status [71]. Given its adaptive nature, we use and adapt the Mirzoev and Kane (2017) health systems responsiveness framework (see below) [72]. Responsiveness is observed on both sides of service provision, i.e., by analyzing the experiences of users (individuals, their families, and communities) and providers (service providers, managers, and policymakers) (Fig. 1).

Two determinants affect experiences: users’ expectations (shaped by their characteristics and relationships with their families and communities) and the health systems’ response to these expectations [72]. Expectations are influenced by social representations of what constitutes (poor) health, needs (that are heterogeneous), appropriate services (e.g., multilingual), and appropriate conduct in service provision. These expectations are shaped by the characteristics of the services available (which are based on viability, i.e., organizations’ ability to acquire and maintain its human, material and financial resources in order to adapt services to current and future needs [73]), their quality, and the trust placed in them (interpersonal trust and trust in organizations) [74]. The policymakers–managers–service providers trio highlights the importance of involving all decision-making levels (macropolitical, i.e., provincial/territorial or federal; organizational; individual) in adjusting the response to needs (attention) while ensuring continuity and accessibility of services in the event of a crisis, and while remaining accountable to users [75].

### Document review

First, we will review intersectoral initiatives that have emerged during the pandemic to address the increased and more diverse needs of refugees, asylum seekers, and migrants without status. We will conduct a document review [76] to compile an inventory of intersectoral initiatives that have emerged or expanded as a result of the pandemic in Montreal, Sherbrooke, and Toronto, and are previously analyzed, reported on, and/or studied by co-investigators, collaborators, and colleagues. The document review will consist of both peer-reviewed and gray literature. However, given the fact that most of the (primarily, organizational) literature is not peer-reviewed, the literature review will not extensively focus on peer-reviewed documents, but rather on gray literature. To carry out this document review, we will use three sources. We will search Google Scholar for peer-reviewed literature, and gather relevant gray literature through our knowledge users’ website documentation, and through soliciting experts’ advice (members of our advisory boards and members of our research team). We expect the latter to be the major source of documentation. We expect documentation to be composed of peer-reviewed and non-peer-reviewed reports, as well as organizational and routinely collected documents by our partners. Data extraction will be based on an analysis grid in an Excel spreadsheet (available upon request), featuring the following categories—objectives; target population; key players; governance and coordination tools; implementation challenges; key results/successes of the initiative; challenging owing to long-term sustainability. This will enable us to: (a) account for the differences between Quebec and Ontario in terms of the structures and operations of public organizations and CBOs in a crisis context, and (b) provide a more in-depth level of detail on the content of diverse initiatives documented in the literature. The initiatives will be classified into two main categories, namely: intersectoral initiatives aiming to prevent and control COVID-19 (such as vaccination outreach strategies and mobile testing clinics), and intersectoral initiatives addressing the consequences of COVID-19 policies (e.g., lockdown policies and physical distancing) for refugees, asylum seekers, and migrants without status (such as local crisis units, food security interventions, interventions to reduce social isolation, etc.). The information that will be gathered may also offer insightful information as to whether and how these initiatives accounted for the differentiated needs of these three populations. This background information will serve as a basis upon which we will then build an empirical analysis.

### Study design

A qualitative multiple case study design will be used [77]. An individual case will be an intersectoral initiative selected through deliberative process (see research activity 1 below). Each case will be considered comparatively across the various selected initiatives (i.e., cases). Qualitative research methods are the most appropriate for identifying the relational, institutional, and contextual dimensions of the phenomena under study [78].

### Data collection

Each research activity will be conducted in both French and English, to reflect Canada’s language diversity and the language preferences of our participants. Other languages will be used to recruit and conduct focus groups with refugees, asylum seekers, and migrants without status to allow our participants to engage in the research in the language they are the most comfortable speaking and understanding.

#### Research activity 1

Findings from the document review will be shared with advisory board members, as well as community organizers of the public health network in Quebec and Toronto Public Health in Ontario. After reaching a consensus among these key actors, regarding the inclusion and exclusion criteria to be applied, these persons will be invited to participate in deliberative workshops [79, 80] to (a) comment on the inventory and (b) reach consensus on intersectoral initiatives identified for each category. This activity may also lead to a revision of our initial list. Two deliberative workshops (virtual or face-to-face, depending on the COVID-19 measures in place at this stage in the research) are planned: one in Quebec and one in Ontario. Approximately 30–40 people (advisory board members and community organizers, and additional experts if needed) will participate in these deliberative workshops. Observational notes will be taken during the workshops, to record the processes and discussions that lead to the deliberation and initiative selection.

#### Research activity 2

Based on the findings from the first research activity, we will analyze selected initiatives from the point of view of service providers, by documenting the implementation processes of these initiatives in the pandemic context. These interviews (face-to-face or by video conference) will be conducted in neighborhoods of Montreal (*n* = 30), Sherbrooke (*n* = 20), and Toronto (*n* = 30) with (1) policymakers, frontline workers, and managers from public organizations (e.g., managers of healthcare services, health professionals) and (2) with managers, frontline workers, and volunteers of CBOs. Fewer interview participants are planned for Sherbrooke because of the city’s smaller size in comparison with Montreal and Toronto. The selected neighborhoods in the three cities, which have high volumes of refugees, asylum seekers, and migrants without status, are the following: Montréal-Nord, Côte-des-Neiges, and Sud-Ouest in Montreal [81]; Ascot and Jardins Fleuris in Sherbrooke [82]; North York and Downtown Toronto in Toronto [83]. A semi-structured interview guide (available upon request) will be used to elicit perspectives on the initiatives’ implementation processes focusing on (a) perceptions of viability, continuity, attention to needs, accessibility, trust, and quality of service delivery for refugees, asylum seekers, and migrants without status and (b) the challenges and opportunities of collaborative work [72]. Advisory board members will review and comment on this guide.

#### Research activity 3

Third, using a focus group discussion guide (also available upon request), we will explore experiences with selected intersectoral initiatives from the point of view of refugees, asylum seekers, and migrants without status, who have arrived in the past 5 years, and who used the services offered through these initiatives versus those who did not have access to these services. Specific strategies will be implemented to encourage the participation of hard-to-reach groups, based on lessons learned from previous research carried out in Montreal [84]. As research collaborators of RÉAC!, ten refugee and asylum seeker representatives (four in Toronto, four in Montreal, two in Sherbrooke) will receive compensation to help us identify strategies to reach these categories of migrants. Research assistants (RAs) from diverse backgrounds and who are multilingual will be hired to facilitate recruitment and moderate focus groups (see below). Together with our research partners, we will identify the locations of these prospective participants so that RAs can recruit them in person (if circumstances permit) or through resource persons in their neighborhood. In total, about 80 refugees, asylum seekers, and migrants without status will be recruited to participate in focus groups (i.e., 30 in Montreal, 20 in Sherbrooke, and 30 in Toronto). A public communications strategy will be used in combination with purposive and snowball sampling methods to recruit participants. Focus group discussions will be organized by gender and language categories [85]. Focus group facilitators will be chosen on the basis of sociodemographic criteria, being mindful of gender equality. For example, women facilitators will facilitate focus groups with women. Focus group facilitators will closely work with the project’s partners for recruiting experienced interpreters.

#### Research activity 4

Fourth, findings from research activities 2 and 3 will inform an open discussion on remaining challenges related to the implementation, scaling-up, sustainability, and/or institutionalization (i.e., these initiatives being codified in action plans and policy frameworks) of the selected intersectoral initiatives. Discussion forums (face-to-face or remote) with managers and stakeholders of CBOs and public organizations who have expressed an interest in participating in these forums (either as research participants or as knowledge users who attended our findings dissemination workshops), and the knowledge-user members of advisory boards, will enable the co-production of factsheets and infographics (detailing the content of each initiative, implementation processes, and sustainability challenges). Two forums per province will be organized.

### Data analysis

#### Research activity 1

Observational notes and outcomes of deliberative workshops will be added to datasets for research activities 2 and 3 and analyzed in the same way. We expect a selection of approximately 20 initiatives in total (across the three cities and reflecting the two aforementioned categories of intersectoral initiatives).

#### Research activities 2 and 3

Data will be transcribed, then coded using QDAMiner, and analyzed using thematic analysis [86]. The components of the adapted framework by Mirzoev and Kane will inform our main thematic categories, paying particular attention to the identification of co-evolution processes within intersectoral collaboration mechanisms, and in the context of the pandemic. A deductive–inductive approach will be employed and informed by the aforementioned framework while also leaving the possibility for empirical data to bring out additional themes and subthemes. In findings interpretation, we will highlight differences and similarities across the three cities and across the selected initiatives. In addition, we will provide key information on the initiatives’ capacity to respond to the differentiated needs of refugees, asylum seekers, and migrants without status. Our analysis will also seek to (a) identify themes related to the holistic needs of our study population, including their social determinants of health, prior to and during the pandemic, and (b) reflect on whether the COVID-19 crises exacerbated some of the pre-existing issues.

#### Research activity 4

The data collected from discussion forums will be synthesized and reported using factsheets that will be made available to CBOs, public organizations, and policymaking bodies. On the basis of issues identified in these forums, we will also adapt a monitoring tool based on the Pluye and Ridde model [87]. This model proposes to evaluate the elements that are favorable or unfavorable to sustainability and/or implementation (e.g., stabilization of organizational resources, incentives or benefits for the actors) on a regular basis, and it will be adapted to the needs of partners.

### Ethical considerations

Refugees, asylum seekers, and migrants without status participating in the research will receive compensation. Applications for ethics approval were granted by the ethics review boards of the University of Toronto and the University of Montreal, as well as participating *Centres intégrés universitaires de santé et de services sociaux* (CIUSSS). Conducting studies with underserved populations requires going beyond mere procedural ethics. Thus, we will account for relational ethics in order to ensure mutual respect and create links between researchers and participants [88]. In addition, a list of mental health and wellbeing support tools will be shared with all focus group discussion participants.

## Discussion

### Feasibility and limitations

Access to key informants and users will be facilitated by resource persons in each of the research partner organizations that have agreed to collaborate. The research team’s previous relationships with several project collaborators, as well as ongoing projects with public service providers, are key entry points to accessing data about intersectoral initiatives and participant recruitment.

The limitations related to this research are twofold. First, access to refugees, asylum seekers, and migrants without status, may remain difficult as the pandemic continues. Indeed, these population categories were already considered hard to reach for research purposes. The pandemic may have made access to these persons even more complex; we foresee even more challenges than usual in recruitment. Our research partners will help mitigate this issue. For instance, our collaboration representatives of refugees and asylum seekers in all three cities will help locate the neighborhoods where prospective participants live (including migrants without status), and identify the most adequate ways to approach them [84]. We will also ask our community partners to share information about our study with their client listservs and through identifying persons that use their services that they think would be ideal for us to speak with (and who would be interested in participating), and then sharing the information about our study directly with them. Second, we will closely monitor the evolution of COVID-19 and its potential impact on the research. The research team will meet each month to assess the situation, adjusting our plans in strict compliance with public health measures. We will thus collect data and hold research meetings using either adequate equipment to maintain physical distancing, or videoconferencing and other remote communication tools. However, using these tools may incur difficulties for people with digital literacy issues, particularly in the case of focus groups. This will require focus group facilitators’ attention, notably in supporting the prospective participants’ technical abilities. In addition, the lives and day-to-day operations of research participants might also be affected. Confronted with a crisis and/or chronic staffing shortages, frontline workers may be less available for interviews. Pre-existing trust relationships with research partners will support the development of consensual contingency plans. Lastly, issues of recruitment in the context of COVID-19 will highlight the relevance of conducting research on providing adequate responses to the needs of refugees, asylum seekers, and migrants without status in times of crisis.

### Knowledge translation plan

Building on the participatory governance of our research project, we include diverse knowledge translation (KT) strategies which aim to: (1) engage knowledge users (refugees, asylum seekers, frontline workers, managers, policymakers) in the different stages of the research, (2) disseminate study findings owing to the most promising initiatives from a health systems responsiveness perspective to strengthen the capacities of public and community frontline workers and managers, and (3) support the decision-making processes of policymakers and funders.

First, the members of our two advisory boards (one in each province), will be meeting four times a year and will make recommendations at key stages of the research, (e.g., on “winning” recruitment strategies and the planning of deliberative workshops). By commenting on preliminary findings presented at advisory board meetings, members will also contribute to analyses.

Second, at the end of the research, several KT publications will be designed and disseminated, namely: (a) multilingual factsheets for refugees, asylum seekers, and migrants without status, presenting the key findings of this research, including infographics; (b) a KT guide, as in Ref. [89], including factsheets listing promising initiatives and infographics aimed at all those involved in the care and orientation of refugees, asylum seekers, and migrants without status (CBOs and public service providers); (c) policy briefs for policymakers (at all levels: municipal, regional, provincial, national level) and philanthropic foundations.

Third, toward the end of the research project, findings will also be discussed and validated with policymakers (Public Health Agency of Canada, Quebec’s relevant ministries, Ontario Health, City of Toronto, City of Montreal, City of Sherbrooke) during dissemination workshops held in each province (with approximately 15 participants per workshop).

Lastly, a final symposium, also involving researchers from other provinces and their own knowledge-user networks, will bring visibility to this research’s findings across Canada and provide the opportunity to expand the growing knowledge-exchange network of frontline workers supporting refugees, asylum seekers, and migrants without status beyond Quebec and Ontario. Findings will also be shared via international conferences and peer-reviewed publications that will be either open access or made openly available through a repository.

### Expected contributions

COVID-19 provides us with an unprecedented opportunity to study intersectoral collaborations. In 2020 and 2021, Canada saw a unique surge in intersectoral initiatives to respond to increasingly diverse population needs. This research focuses on refugees, asylum seekers, and migrants without status who are more likely than Canadian citizens and economic immigrants to be affected by COVID-19 and the decisions implemented to mitigate its spread and community impacts. To ensure research uptake and impact, we will apply a unique participatory and inclusive approach, which involves bilingual research activities, multilingual participant recruitment, refugees’, asylum seekers’, and frontline workers’ engagement in the research’s governance.

At individual and organizational levels, our theory-informed findings will provide in-depth analyses of the implementation processes of intersectoral initiatives for frontline workers and managers in times of crisis and beyond. The number and needs of refugees, asylum seekers, and migrants without status is expected to continue to grow beyond the pandemic. Our results will highlight the opportunities for community–public linkages to sustain and strengthen intersectoral coordination and improve services for these underserved populations. There is indeed too little evidence about what intersectoral initiatives or forms of collaboration could be continued over time, beyond the crisis context, and how to sustain and scale these initiatives. Our research will also highlight the ways through which intersectoral action can provide differentiated yet equitable and culturally sensitive services to all three population categories (refugees, asylum seekers, and migrants without status), in the context of a pandemic and beyond.

At the macropolitical level, the contextual analyses and the comparison between the two provinces will allow us to show how differences in the organization of services between Ontario and Quebec influence intersectoral action and the innovations produced. Our results could thus contribute to strengthening actions taken by CBOs and public service providers, which requires an institutional linkage that goes well beyond the context of the pandemic, and well beyond the local context.

Beyond the advancement of knowledge, our approach will build the capacity among: (1) refugees and asylum seeker representatives who will sit on our advisory boards after being trained in public’s participation in a research project, and (2) stakeholders participating in advisory boards. This research could eventually serve to develop a community of practice between community and public organizations, potentially facilitated by employees of federal partner organizations in each province.

Second, the ongoing involvement of representatives of managers and policymakers (at all levels of government) will help to disseminate the promising practices identified and influence decision-making by federal and provincial/territorial government authorities. In particular, our links with the Public Health Agency of Canada and the Ministry of Immigration, Frenchisation and Integration in Quebec, will help facilitate links with public authorities and knowledge translation at the highest political level. As health crisis contexts will continue to shape our lives in the future [90], decision-makers would benefit from relying more on intersectoriality, and particularly CBOs’ experience with providing responsive, well-funded, and culturally acceptable care to these vulnerable populations.

## Data Availability

Not applicable.

## Footnotes

1 Because we foresee numerous barriers to the ongoing engagement of migrants without status throughout the 2 years of the research project, we collectively decided to include refugees and asylum seekers only, in our advisory boards.
